# Two-Dimensional Cellular and Three-Dimensional Bio-Printed Skin Models to Screen Topical-Use Compounds for Irritation Potential

**DOI:** 10.3389/fbioe.2020.00109

**Published:** 2020-02-21

**Authors:** Zhengxi Wei, Xue Liu, Masato Ooka, Li Zhang, Min Jae Song, Ruili Huang, Nicole C. Kleinstreuer, Anton Simeonov, Menghang Xia, Marc Ferrer

**Affiliations:** ^1^Division of Pre-Clinical Innovation, National Center for Advancing Translational Sciences, National Institutes of Health, Rockville, MD, United States; ^2^3D Bioprinting Core, National Eye Institute, Bethesda, MD, United States; ^3^Division of the National Toxicology Program, National Institute of Environmental Health Sciences, National Institutes of Health, Research Triangle Park, NC, United States

**Keywords:** skin irritation, bio-printing, reconstructed human epidermis, full thickness skin tissue, skin sensitization, Toxicology in the 21st Century, high throughput screen

## Abstract

Assessing skin irritation potential is critical for the safety evaluation of topical drugs and other consumer products such as cosmetics. The use of advanced cellular models, as an alternative to replace animal testing in the safety evaluation for both consumer products and ingredients, is already mandated by law in the European Union (EU) and other countries. However, there has not yet been a large-scale comparison of the effects of topical-use compounds in different cellular skin models. This study assesses the irritation potential of topical-use compounds in different cellular models of the skin that are compatible with high throughput screening (HTS) platforms. A set of 451 topical-use compounds were first tested for cytotoxic effects using two-dimensional (2D) monolayer models of primary neonatal keratinocytes and immortalized human keratinocytes. Forty-six toxic compounds identified from the initial screen with the monolayer culture systems were further tested for skin irritation potential on reconstructed human epidermis (RhE) and full thickness skin (FTS) three-dimensional (3D) tissue model constructs. Skin irritation potential of the compounds was assessed by measuring tissue viability, *trans-*epithelial electrical resistance (TEER), and secretion of cytokines interleukin 1 alpha (IL-1α) and interleukin 18 (IL-18). Among known irritants, high concentrations of methyl violet and methylrosaniline decreased viability, lowered TEER, and increased IL-1α secretion in both RhE and FTS models, consistent with irritant properties. However, at low concentrations, these two compounds increased IL-18 secretion without affecting levels of secreted IL-1α, and did not reduce tissue viability and TEER, in either RhE or FTS models. This result suggests that at low concentrations, methyl violet and methylrosaniline have an allergic potential without causing irritation. Using both HTS-compatible 2D cellular and 3D tissue skin models, together with irritation relevant activity endpoints, we obtained data to help assess the irritation effects of topical-use compounds and identify potential dermal hazards.

## Introduction

Skin provides a physical barrier to protect the body from environmental insults, including chemical agents (Liu et al., 2016). In the context of regulatory hazard classification (Safety and Administration, 2012), chemicals that cause reversible local skin tissue damage upon dermal exposure are defined as skin irritants. Assessment of skin irritancy is a regulatory requirement in the safety evaluation of industrial and consumer products. Traditionally, irritation potential is evaluated by the Draize test, an acute toxicity test used by the FDA (Draize et al., 1944), which applies a patch containing the test substance directly to rabbit skin. In consideration of animal welfare, in the last decade, the cosmetics industry in the EU, Israel, India, Norway, Turkey, Australia, and New Zealand have been mandated to use RhE tissues for evaluating skin irritation and corrosion potential of cosmetics ingredients and products as an alternative to animal testing (OECD, 2004, 2015; Tornier et al., 2006; Co-operation and Development, 2013). Most RhE skin equivalents are tissues made with keratinocytes that model a stratified epidermis, but have the following drawbacks: (1) these models do not have the sample throughput needed for large scale profiling of compounds at different doses; and (2) they do not reproduce the physiological complexity found in human skin tissue, including the lack of cell-cell interactions between keratinocytes and fibroblasts in the dermis layer. These cell-cell interactions are important for the normal function of skin as a physical barrier for the body, including formation of epidermal-dermal junction, epidermal differentiation, and stratification (El Ghalbzouri et al., 2005; Wojtowicz et al., 2014). Their absence in a cellular model of the skin can result in the lack of immunological responses relevant to irritation effects caused by compounds (El-Ghalbzouri et al., 2002; Hänel et al., 2013; Sriram et al., 2015). Therefore, there is a need for a platform of cellular assays that enables the large-scale screening of compounds while producing data that are relevant to and predictive of irritation responses in humans.

*In vitro* cellular models for large scale drug testing currently rely on 2D cellular monolayers because of practical considerations and ease of implementation. For example, high batch-to-batch and well-to-well reproducibility and robust changes in assays are critical for HTS. However, these 2D cellular models have low physiological relevance and limited clinical predictive value. In spite of their low predictability of *in vivo* irritation responses, 2D cellular models can provide a first indication to prioritize the test substances to determine whether compounds will have toxic effects *in vivo*. In our strategy, a platform of cellular assays was developed to help predict the skin irritation potential of topical-use chemicals. We first used a cytotoxicity assay with keratinocytes grown in 2D monolayer. Compounds that were active in these assays were then further tested for irritation activity using biofabricated 3D skin tissue models. The recent advances in tissue biofabrication techniques, including the use of bioprinting technologies, enables the reproducible production of biofabricated biological tissues (Lee et al., 2013; Ng et al., 2018). It has been shown that bioprinting allows for the controlled formation of layered 3D skin tissues in a multiwell plate format (Derr et al., 2019). The bioprinting protocols are very versatile so that additional physiological complexity can be included to more closely mimic native human skin and create a more physiologically relevant assay system for compound testing.

In this study, several assay readouts were developed with both of the 3D models, RhE and FTS, including cell viability, TEER, and the secretion level of IL-1α and IL-18 which are relevant to irritation and sensitization skin responses. TEER is commonly used to measure the tight junction integrity of an epithelial monolayer and assess skin barrier function (Srinivasan et al., 2015). Damage produced by both irritants and sensitizers on keratinocytes in the epidermis is associated with release of IL-1α as a primary defense event (Galbiati et al., 2014; Worm, 2014). It has been shown that RhE models recapitulate this response when treated with irritants (Poumay et al., 2004). Keratinocytes also produce IL-18 when exposed to irritants (Companjen et al., 2000). The cleavage and release of IL-18 has been used as a biomarker to distinguish sensitizers from irritants in RhE models (Corsini et al., 2009). Thus, the IL-1α and IL-18 secretion levels in RhE and FTS models were used to assess the irritation potential of selected topical-use compounds.

**FIGURE 1:**
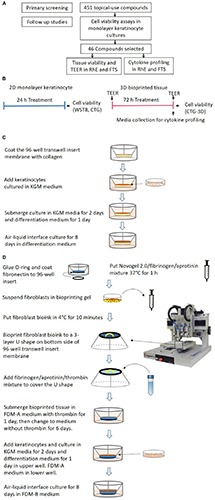
**(A)** A flowchart for the identification of potential irritants. **(B)** A schematic signifying the comparison of test timelines and endpoints between the 2D monolayer culture and 3D bio-fabricated tissues. **(C)** A diagram of the generation of the bio-fabricated RhE model. **(D)** A schematic drawing of the bio-printing method for the FTS model.

The ability of the proposed assays as a screening tool to quickly and efficiently test environmental chemicals for their skin irritation potential was assessed by implementing a chemical library screen. We selected 451 topical-use compounds which included 55 OECD reference substances and 396 topical-use chemicals from the Tox21 10K chemical library. The 451 compounds were first tested by using a cell viability assay of keratinocytes grown in a 2D monolayer, enabling quick detection of potential irritants. Of the 451 compounds tested, 46 were further evaluated in the validated biofabricated RhE and FTS models developed in 96-well plate format. Measurement endpoints included tissue viability, TEER, and cytokine secretion analysis, which enabled determination of the irritation potential of compounds as well as potential sensitization effects. The workflow of this study is summarized in Figures 1A,B. The platform identified known irritants and also allowed us to distinguish the potential sensitizer activity of some compounds.

## Materials and Methods

### Compound Library

The Tox21 10K chemical library consists of approximately 10,000 (∼8300 unique) small molecules including pesticides, drugs, industrial chemicals, and food additives, commercially sourced by the NTP, NCATS, and EPA (Attene-Ramos et al., 2013; Tice et al., 2013). Skin related chemicals such as topical-use drugs, cosmetic ingredients, and pesticides with dermal exposure risk were selected as a subset library for this study. Also, references chemicals in OECD’s test guidelines: 404, 430, 431, 435, and 439 (Griesinger et al., 2009) were included.

### Cell Line and Culture Conditions

Human primary neonatal keratinocytes and KGM were purchased from Sciencell (Catalog number of the cell: 2100, catalog number of the media: 2101, Carlsbad, CA, United States). hTERT and SV40 early region immortalized NHEK were purchased from Evercyte (Vienna, Austria). The cells were cultured in KGM-2 Bullet Kit (Lonza, Walkersville, MD, United States) without adding GA-1000. Neonatal human dermal fibroblast (NDF) were purchased from the ATCC (Catalog number: PCS-201-010, Manassas, VA, United States). Fibroblasts were maintained in minimal essential medium (MEM) supplemented with 5% FBS (Thermo Fisher Scientific, Waltham, MA, United States). The cells within passage 3–4 were used. All the cells were maintained at 37°C under a humidified atmosphere and 5% CO_2_. All the cultures were routinely monitored for mycoplasma contamination using MycoAlert^TM^ PLUS mycoplasma detection kit (Lonza, Walkersville, MD, United States).

### Cell Viability Assays in Human Primary Neonatal Keratinocytes and Normal Human Epidermal Keratinocytes

Human primary neonatal keratinocytes and normal human epidermal keratinocytes were seeded at 2000 cells/well/4 μL of culture medium into 1536-well black wall, clear bottom plates (Greiner Bio-One, Monroe, NC, United States) using a Multidrop^TM^ Combi (Thermo Fisher Scientific). The cells were incubated at 37°C for 5 h for attachment. Then 23 nL of the compounds were transferred to the assay plates to reach final concentrations from 0.4 nM to 92 μM. The cells were treated for 24 h.

The cell viability of human primary neonatal keratinocytes and normal human epidermal keratinocytes was measured by using a Cell Counting Kit (WST8, Dojindo, Rockville, MD, United States) multiplexed with CellTiter-Glo^®^ Luminescent Cell Viability Assay (Promega, Madison, WI, United States). Tetra-octylammonium bromide was the positive control for data normalization. After compound treatment for 22 h, 1 μL WST8 reagent was added into each well. The plates were incubated at 37°C for 2 h before measuring absorbance at 450 nm by PHERAstar^TM^ microplate reader. After measuring absorbance reading, 4 μL CellTiter-Glo^®^ reagent was added to each well. The luminescence signal was measured with a ViewLux^TM^ plate reader after a 30 min incubation at room temperature.

### Generation of RhE Model

RhE was generated as previously described (Smits et al., 2017). A schematic of the process is shown in Figure 1C. In brief, HTS-96 transwell inserts with 0.4 μm pore size polycarbonate membranes (HTS Transwell-96, 3381, Corning, NY, United States) were treated with 50 μL of rat tail collagen (100 μg/mL) in (PBS, Corning, NY, United States) for 1 h at room temperature. The collagen solution was kept cold before usage. The transwell insert was then rinsed once with 100 μL of sterile cold PBS. Fifty μL of a human neonatal primary keratinocytes suspension (2 × 10^5^ cell/cm^2^ in KGM media) were seeded by pipetting on top of the membrane of the transwell insert. The cells were cultured submerged in 50 μL KGM media for 2 days. Afterward, the media was changed to keratinocyte differentiation media for another 24 h. The keratinocyte differentiation media consists of CnT-PR-3D media (Zen-Bio, Research Triangle Park, NC, United States) and Dulbecco’s Modified Eagle Medium (DMEM, Thermo Fisher Scientific) with a 3:2 ratio. The tissues were then cultured in an ALI with 400 μL of keratinocyte differentiation medium in the base plate well, with no media in the transwell, for 8 days. The transwell insert was placed on a custom lifter (6 mm height), between the base plate and the insert tray, in order to add enough volume of media in the base plate well. The culture medium was changed every other day. All the cultures were routinely monitored for mycoplasma contamination using a MycoAlert^TM^ PLUS mycoplasma detection kit (Lonza).

### Bio-Printing of 3D Human Dermis in 96-Well Plate

The schematics for the bio-printing of the dermis are shown in Figure 1D. A custom 3D-printed polycarbolactone (PCL) O-ring (outside diameter 6 mm, inside diameter 5 mm, height 700 μm) was glued with bio-friendly silicon glue (Kwik-cast sealant, World Precision Instrument, Sarasota, FL, United States) onto the bottom of each well of a Corning^®^ HTS Transwell^®^ plate -96 Permeable Supports with 1 μm polyester pore size membrane (Catalog number 3381, Corning, NY, United States). The O-ring provided a sealed wall around the dermis compartment. The membranes of the transwell inserts were coated with 50 μL of fibronectin solution (0.03 mg/mL in distilled water) for 1 h at room temperature the night before printing. A mixture of Novogel 2.0 (60 mg/mL, Organovo, San Diego, CA, United States), fibrinogen (2.5 mg/mL) and aprotinin (0.075 unit/mL in DPBS, Sigma-Aldrich, St. Louis, MO, United States) was prepared as bioprinting hydrogel. On the day of printing, the prepared bioprinting hydrogel was kept in a 37°C water bath for 1 h before use. Neonatal fibroblasts were suspended with 1 mL printing hydrogel at a concentration of 8 × 10^6^ cells/mL in a 2.5 mL syringe (RegenHU, Villaz-Saint-Pierre, Switzerland). The cell mixture was refrigerated at 4°C for 10 min to allow gelation before mounting on to the bioprinter (RegenHU, Villaz-Saint-Pierre, Switzerland). A 3-layer U-shaped pattern was bioprinted with the cell/hydrogel mixture, with a total volume of 5 μL/transwell, onto the bottom side of the transwell insert membrane. This structure was covered with 25 μL fibrinogen (4.5 mg/mL), aprotinin (0.075 unit/mL), and thrombin mixture (1 unit/mL) solution in DPBS using a pipet. The freshly bioprinted tissues were kept at room temperature for 15 min before adding 200 μL of fully supplemented dermal medium A (FDM-A; supplemented components in Supplementary Table 3) with 1 unit/mL thrombin in the basal side. This hydrogel mixture provided a proper ECM for the fibroblasts to migrate. The cells in the printed structure together with the added hydrogel formed the 3D structure of the dermis. Fifty μL of the same FDM-A media supplemented with thrombin was then added in the apical side. The tissues were incubated at room temperature for 2 h and moved to the 37°C with 5% CO_2_, 95% humidity incubator afterward. The tissues were maintained in FDM-A supplemented with thrombin for 24 h. The media was then switched to FDM-A without thrombin and incubated for another 6 days. The medium of tissues cultured in the base plate and transwell was changed every other day.

### Generation of FTS Model

The schematics for the generation of FTS are shown in Figure 1D. On day 8 of dermis tissue incubation, 50 μL of a human neonatal primary keratinocytes suspension (2.0 × 10^5^/cm^2^ in KGM) were pipetted into the apical side of the transwell insert while the bioprinted dermis remained on the bottom side. The transwell insert was rested on a custom lifter (6 mm height) between the base plate and the insert tray in order to leave enough room for the ALI step. The tissue was incubated with 450 μL FDM-A medium (see Supplementary Table 3) in the base plate well and 100 μL KGM medium in the apical insert well. After 48 h, the apical medium was changed to differentiation medium (see section “Generation of RhE Model”) for another 24 h. Then the tissue was ALI cultured for 8 days with 350 μL FDM-B medium (see Supplementary Table 3) in the base plate well compartment and no media in the apical insert. All the cultures were routinely monitored for mycoplasma contamination using MycoAlert^TM^ PLUS mycoplasma detection kit (Lonza, Walkersville).

### Tissue Embedding and Cryosectioning

Reconstructed human tissues were fixed in 4% paraformaldehyde overnight at 4°C. The tissue was washed in PBS three times for 15 min each. The tissue was then taken through a gradient of 15% and 30% weight/volume sucrose in PBS. Whole tissues were removed from sucrose, blotted dry, and embedded in Tissue-Tek CRYO-OCT Compound (Andwin Scientific, Tryon, NC, United States). Blocks were stored at −80°C, sectioned on a Leica CM3050 S cryostat into 12 μm thick sections, and placed on SuperFrost^TM^ Plus slides (Thermo Fisher Scientific).

### Immunostaining and Imaging

The immunostaining of the tissue was prepared using fully automated IHC machine (BOND RXm, Leica Biosystems, IL, United States). Tissue sections were incubated for 3 min with 1X Leica Bond Wash solution (10X Bond wash was diluted using deionized water, Leica Microsystems, Catalog number AR9590) and blocked for 20 min with 2% normal goat serum in PBS. The slides were incubated with primary antibodies (dilution factor and resources in Supplementary Table 3) at 150 μL/slide using Leica Microsystems M211518 for 1.5 h, followed by three washes with 1X Leica Bond Wash solution at room temperature. Thereafter, the slides were incubated with secondary antibodies at room temperature with 1:2000 Hoechst for 20 min followed by three washes with 1X Leica Bond Wash solution. Images of the slides were captured using Leica TCS SP8 MP multiphoton microscope with a 25x water objective lenses and processed using Leica LAS X software.

For H&E staining, sections were placed at room temperature for 10 min. The sections then underwent standard H&E (Thermo Fisher Scientific) staining. Brightfield photographs (20X or 40X) were made with an EVOS^®^ Life technology microscope.

### Transepithelial Electrical Resistance Measurements

Transepithelial electrical resistance measurements were acquired from the ALI on day 8 using an automated TEER measurement system (World Precision Instruments, Sarasota, FL, United States). The transwell was filled with 100 μL DPBS in the apical region. The contribution of the PET membrane was measured and subtracted from the sample values. TEER final values in Ω^∗^cm^2^ were obtained by multiplying the electrical resistance with the skin surface area. Any tissue with a TEER value lower than 500 Ω^∗^cm^2^ was not used in this research (Matsusaki et al., 2015).

### Cell Viability in RhE and FTS Models

Tissue viability was measured by using CellTiter-Glo^®^ 3D (CTG-3D) luminescent cell viability assay (Promega). CTG-3D was mixed with DPBS at 1:2 volume to volume ratio; 100 μL of the diluted CTG-3D was added to the upper compartment of each transwell and 200 μL of the diluted CTG-3D was added to the lower compartment. The tissues were incubated at 37°C for 30 min before harvesting by puncturing the tissues with 20 μL pipet tips. The whole plate was shaken on a rocker for 30 min at room temperature to mix the liquids in the upper and lower compartments. Fifty μL of the liquid was transferred to white solid 96-well plates and the luminescence activity was read by Viewlux^TM^ plate reader.

### Measurement of Secreted Cytokine Level

The medium from each well with tissue was collected before tissue harvesting and stored at −80°C in aliquots. According to the U-PLEX manufacturer’s protocol, antibodies against IL-1α and IL-18 were coated onto MSD 96-well plates and the plates were shaken at room temperature for 1 h. Fifty μL of experimental samples and cytokine standards were added to each well after washing off the excessive unbound antibodies with PBS and Tween 20^®^ (PBST; 0.05% Tween 20^®^) three times. Detection antibody was added to each well after shaking the whole plate on an orbital shaker at the speed of 700 rpm/min overnight at 4°C. Each well was washed three times with 0.05% PBST before adding 2X reading buffer. Electrochemiluminescence was measured using a MESO QuickPlex SQ 120 reader.

### Data Analysis

#### Monolayer Cell Viability Data Analysis

Data normalization and concentration-response curve fitting for the data from the qualitative high throughput screen (qHTS) and follow up studies were performed as previously described (Huang, 2016). Briefly, raw plate reads for each titration point were first normalized relative to the positive control compound and DMSO-only wells (Benzalkonium chloride = −100%, DMSO = 0%) as follows:% Activity = [(V_compound_ – V_DMSO_)/(V_pos_ – V_DMSO_)] × 100, where V_compound_ denotes the compound well values, V_pos_ denotes the median value of the positive control wells, and V_DMSO_ denotes the median values of the DMSO-only wells, and then corrected by applying an NCATS in-house pattern correction algorithm using compound-free control plates (such as, DMSO-only plates) at the beginning and end of the compound plate stacks. Concentration-response titration points for each compound were fitted to a four-parameter Hill equation yielding concentrations of half-maximal inhibitory activity (IC_50_) and maximal response (efficacy) values.

#### Tissue Viability Data Analysis

The luminescence activity was normalized to the fold of change over 1% DMSO (vehicle control). The means and SDs were calculated by three replicate plates and reported as fold-change over vehicle control.

#### Tissue TEER Data Analysis

Raw reads of the TEER value (Ω^∗^cm^2^) for tissue were reported for the 46 compounds in 3 replicate plates. The means and SDs of TEER values in concentration-response curves of selected compounds were normalized to 1% DMSO and reported as fold of change over vehicle control.

#### Cytokine Data Analysis

Interleukin 1 alpha and IL-18 cytokine level of each well was calculated according to the standard curves by DISCOVERY WORKBENCH Software v 4.0 (Meso-Scale Discovery, Rockville, MD, United States). The relative increase or decrease of cytokine levels was normalized to the fold-change over 1% DMSO (vehicle control). The means and SDs were calculated by three replicate plates and reported as fold-change over vehicle control.

Statistical analyses were performed using GraphPad Prism software 7.0 (San Diego, CA, United States). Student *t*-test with the Holm–Sidak posttest was used for statistically significant analyses that involved three experimental groups.

## Results

### Cytotoxic Effects of Topical-Use Chemicals in 2D Monolayer Cell Cultures

A total of 396 topical chemicals from the Tox21 10K library and 55 OECD reference chemicals were tested in neonatal primary keratinocytes (NKTC) and immortalized human keratinocytes (NHEK) for cytotoxic activity. Keratinocytes growing as a monolayer were treated with chemicals at 11 concentrations ranging from 100 nM to 92 μM in 1536-well plates for 24 h. Cell viability was then measured using CellTiter-Glo^®^ and WST-8 reagents. The 46 chemicals showing cytotoxicity in both cell lines (efficacy > 50% cell death) were selected and further tested in RhE and FTS models. The IC_50_ and % efficacy values of 46 chemicals are reported in Supplementary Table 1, and the cytotoxic activity of all 451 chemicals is reported in Supplementary Table 4.

### Characterization of Skin Morphogenesis in RhE and FTS Models

**FIGURE 2:**
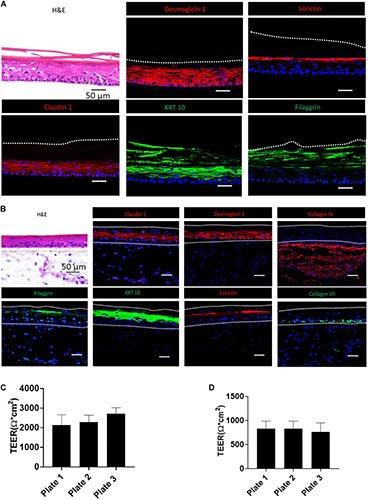
Validation of RhE and FTS models by morphological staining and TEER measurement. **(A)** RhE was harvested at ALI day 8 and cryosectioned or cryopreserved for H&E and biomarker staining of claudin-1, desmoglein-1, filaggrin, keratin-10, and loricrin. **(B)** FTS was harvested at day 21 and cryosectioned or cryopreserved for H&E and biomarker staining of claudin-1, desmoglein-1, collagen IV, filaggrin, keratin-10, loricrin, and collagen VII. **(C)** TEER values, demonstrating barrier function, of RhE models which were replicated in different 96-well HTS plates. **(D)** TEER values of FTS models replicated in different 96-well HTS plates.

H&E and IHC staining were performed to verify the generation of typical human epidermal morphological features for the biofabricated RhE and FTS. One of the critical hallmarks of skin maturation is the formation of a cornified envelop in the SC (Sriram et al., 2018). As shown in Figure 2, H&E staining of RhE displayed the stratified outermost layer, SC, which is a characteristic of normal human epidermis. The appearance of polarized columnar basal keratinocytes and several layers of spinous granular keratinocytes in the H&E staining indicated the formation of the SB and the SG. Desmoglein-1, a calcium-binding transmembrane glycoprotein component of desmosomes in vertebrate epithelial cells, and claudin-1, an important component in tight junctions, were strongly expressed, showing well-developed cell-cell junction in the viable epidermis. Loricrin and filaggrin, localized in the lamellar body of the SG, also showed strong expression and verified the maturation of the RhE. Expression of KRT 10 indicated post-mitotic terminal differentiation of the RhE. Histological staining of the bio-printed FTS sections also demonstrated fully differentiated epidermis with several viable epidermal layers. As shown in Figure 2B, H&E staining highlighted the complex architecture of the FTS for both dermis and epidermis. The formation of the epidermal barrier function was further verified by staining claudin-1 and desmoglein-1 junction proteins that appear in the upper layers of the epithelium. Early differentiation marker KRT-10 indicated an advanced epidermal maturation. The expression of filaggrin and loricrin demonstrated the stratification and cornification of the epidermis. The deposition of basement membrane proteins, collagen IV and collagen VII, showed appropriate anchorage between the epidermis and the dermis.

### Verification of Skin Barrier Function of the Biofabricated RhE and FTS Models

To establish that the biofabricated RhE and FTS models had a matured skin barrier function, the TEER assay, a well-established measurement of the integrity of skin barrier function, was conducted (Abdayem et al., 2015; Niehues et al., 2018). Tissues with TEER values above 500 Ω^∗^cm^2^ were used in the compound screening, for both RhE and FTS models (Figures 2C,D). As shown in Figure 2C, RhE models had TEER values of 2134.9 ± 530.2 Ω^∗^CM^2^ (plate 1, *n* = 60), 2291.1 ± 346.1 Ω^∗^CM^2^ (plate 2, *n* = 60), and 2731.0 ± 294.3 Ω^∗^CM^2^ (plate 3, *n* = 60), which denoted normal skin barrier function. In the FTS models shown in Figure 2D, TEER values were 831.5 ± 156.1 Ω^∗^CM^2^ (plate 1, *n* = 60), 831.7 ± 156.1 Ω^∗^CM^2^ (plate 2, *n* = 60), and 767.5 ± 184.5 Ω^∗^CM^2^ (plate 3, *n* = 58). Noticeably, these values from the FTS models were above 500 Ω^∗^cm^2^, though they were lower than the TEER values from RhE models.

### Effect of Compounds on Cell Viability and TEER in RhE and FTS Models

To assess the responsiveness of RhE to corrosive substances and irritants, we first examined tissue viability with a few compounds categorized as corrosion substances and irritants (Supplementary Figures 1A,B) using the RhE model, as suggested by OECD test guidelines 431 and 439. The benchmark substances, including two corrosives and three irritants from the OECD test guidelines, were tested in triplicate. The relative cell viability was normalized to the PBS-only tissues. Since the application of raw compounds is not possible in an HTS platform, the compounds were dissolved in DMSO and diluted, using PBS, to reach a 200 μM concentration (1% DMSO); they were then added topically for 3 days. We compared our modified protocol (3-day treatment) with the 1 h treatment plus 2-day post incubation protocol from the OECD test guideline 439. As shown in Supplementary Figure 1C, continuous treatment for 3 days did not affect the tissue viability in the vehicle control. Interestingly, the intensive washing steps recommended, in the OECD test guideline 439, appears to decrease the tissue viability, as seen in the 1% DMSO vehicle control wells.

**FIGURE 3:**
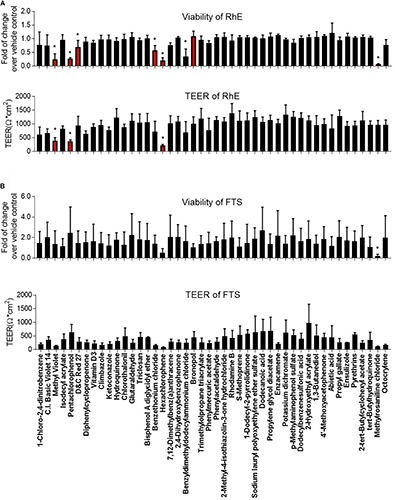
Comparison of viability and TEER in **(A)** RhE and **(B)** FTS models upon treatment of 46 chemicals. The relative increase/decrease of viability level was normalized to the fold of change over 1% DMSO (vehicle control). The mean and SD of viability were calculated by three replicate plates and reported as fold of change over vehicle control. After compound treatment, raw readings of TEER values (Ω^∗^cm2) for three replicate plates of tissues, in each model, were reported. Data are expressed as mean ± SD from triplicate experiments. (*p < 0.05).

We then proceeded to test whether the 46 most cytotoxic chemicals tested in monolayer keratinocytes reduced cell viability in the biofabricated skin tissue models. Seven compounds – pentachlorophenol, methyl violet, D&C red 27, benzethonium chloride, hexachlorophene, benzyldimethyldodecylammonium chloride, and methylrosaniline chloride, – reduced tissue viability in RhE at 200 μM (red bars in Figure 3A and * in Supplementary Table 2). When these chemicals were tested in the FTS model, only methylrosaniline chloride caused a significant reduction in viability (* in Figure 3B and # in Supplementary Table 2). The effects of the compounds on TEER were more pronounced in FTS, but heatmap plots (Figure 5A) showed a higher concordance between TEER and viability in the RhE model (*R*^2^ = 0.33) than for FTS (*R*^2^ = 0.13).

### Effect of Compounds on IL-1α and IL-18 Secretion in RhE and FTS Models

**FIGURE 4:**
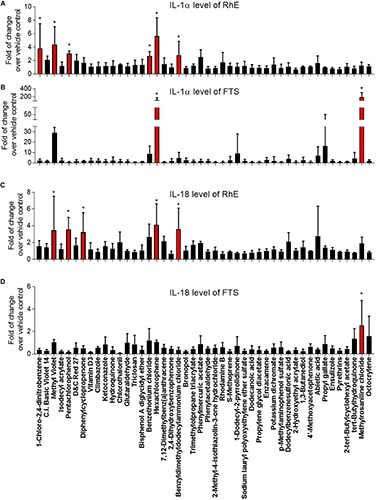
Comparison of secreted IL-1α levels in **(A)** RhE and **(B)** FTS models as well as IL-18 levels in **(C)** RhE and **(D)** FTS models upon treatment of 46 chemicals. The relative increase or decrease of cytokine levels were normalized to the fold of change over 1% DMSO (vehicle control). Data are expressed as mean ± SD from triplicate experiments (*p < 0.05).

**FIGURE 5:**
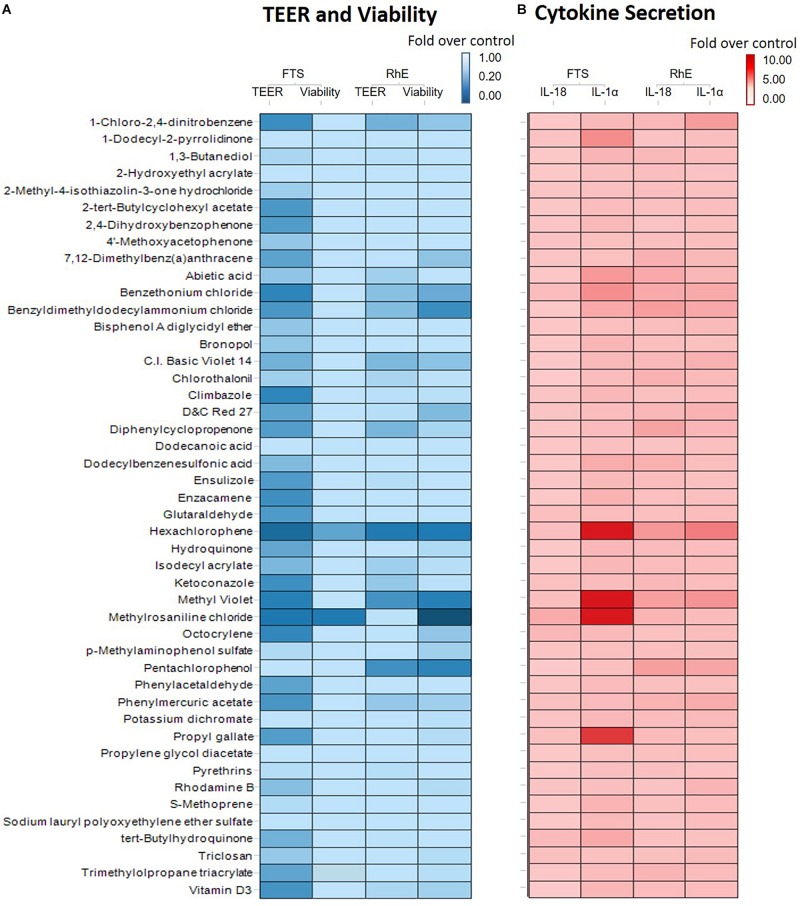
**(A)** Comparison of viability and TEER using RhE and FTS models. The lightest blue color in the gradient represents 100% barrier integrity in the TEER assay and 100% viable tissue in the viability assay, while the darkest blue color signifies 0% barrier integrity (completely disrupted tight junction) in the TEER assay and 0% viability (dead skin). **(B)** Comparison of secreted IL-1α and IL-18 levels in RhE and FTS models. The lightest red color in the gradient denotes no fold change compared with the vehicle control, symbolizing healthy skin. The darkest red color represents a fold change of 10 or more compared with the vehicle control.

Interleukin 1 alpha is a central mediator of innate immunity and inflammation (Corsini and Galli, 1998). Additionally, IL-18 secreted by keratinocytes has been proven to be a biomarker for an allergic response (Corsini et al., 2009; Hänel et al., 2013). To evaluate the potential of a compound to induce irritation and sensitization, we used a multiplex electrochemiluminescence assay to quantify the amounts of IL-1α and IL-18 secretion in RhE and FTS models. As shown in Figure 4A, hexachlorophene, methyl violet, 1-chloro-2,4-dinitrobenzene, pentachlorophenol, benzyldimethyldodecylammonium chloride, and benzethonium chloride significantly induced IL-1α secretion in the RhE model by 5.7-, 4.4-, 3.8-, 3.0-. 2.8-, and 2.7-fold, respectively. Interestingly, methylrosaniline chloride and hexachlorophene significantly increased IL-1α secretion in the FTS model by 193- and 123-fold, respectively (Figure 4B). As shown in Figure 4C, hexachlorophene, benzyldimethyldodecylammonium chloride, pentachlorophenol, methyl violet, and diphenylcyclopropenone induced IL-18 secretion, in the RhE model, by 4.1-, 3.6-, 3.6-, 3.5-, and 3.3-fold, respectively. Methylrosaniline chloride elevated the IL-18 level 2.5-fold greater than the vehicle control in the FTS model (Figure 4D). Interestingly, the IL-1α secretion was more prominent in the FTS model, while IL-18 elevation was more obvious in the RhE model (Figure 5B).

### Concentration-Response Effects of Selected Compounds in FTS Model

**FIGURE 6:**
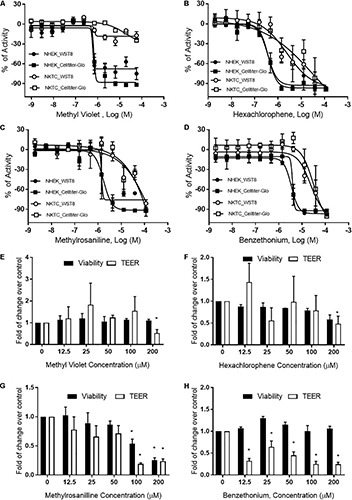
Viability and TEER values of selected chemicals. Cytotoxic effects of **(A)** methyl violet, **(B)** hexachlorophene, **(C)** methylrosaniline, and **(D)** benzethonium in NHEK and NKTC. The viability of each cell type was measured by WST8 and CellTiter-Glo^®^ assays and the percent activity was normalized to the positive control. Concentration-responses of tissue viability and TEER experiments, in FTS, upon treatment of **(E)** methyl violet, **(F)** hexachlorophene, **(G)** methylrosaniline and **(H)** benzethonium. The viability and TEER values were normalized to 1% DMSO (vehicle control). Concentration response curves and bar graphs were expressed as mean ± SD from three biological replicates (*p < 0.05).

**FIGURE 7:**
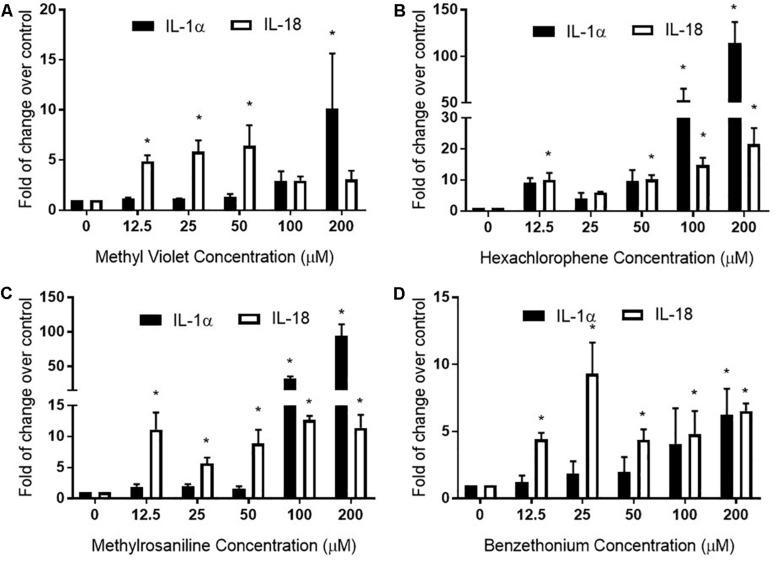
Full thickness skin was treated with **(A)** methyl violet, **(B)** hexachlorophene, **(C)** methylrosaniline, and **(D)** benzethonium in a concentration-dependent manner and the secreted levels of IL-1α and IL-18 were measured. The relative increase or decrease of cytokine levels was normalized to the fold change over 1% DMSO (vehicle control). Concentration-responses were expressed as mean ± SD from three biological replicates (*p < 0.05).

Methyl violet, methylrosaniline, hexachlorophene, and benzethonium which were significantly active in two endpoints compared to vehicle controls, in both RhE and FTS models, were further tested in five concentrations ranging from 12.5 to 200 μM. These compounds had similar IC_50_ values in viability when tested in monolayer keratinocytes (Figures 6A–D) but exhibited different cytotoxicity in FTS (Figures 6E–H) and distinct cytokine secretion profiles (Figure 7). The TEER assay was more sensitive than the viability assay when measuring the integrity of the FTS model; this became evident when 200 μM methyl violet (Figure 6E), 200 μM hexachlorophene (Figure 6F), and benzethonium (Figure 6H) significantly decreased TEER values but did not reduce the tissue viability. Methylrosaniline was the only chemical which significantly reduced TEER and viability. All four of these compounds displayed significant increases in IL-1α and IL-18 secretion levels in a concentration-response manner (Figure 7). Of note, the highest concentrations of each compound tested were able to significantly increase IL-1α secretion. Interestingly, only IL-18 level was increased upon the treatment of low concentrations (12.5–50 μM) (Figure 7).

## Discussion

This study established an integrated monolayer and 3D tissue assay in HTS platform, which enables large-scale dermal toxicology testing. We started with a screen of 451 topical compounds at 11 concentrations using monolayer keratinocyte viability assays. The chemicals showing cytotoxicity in these assays were selected for a single concentration test in bio-fabricated skin tissues. Several dermal toxicants were then selected and studied at multiple concentrations in FTS model. Our results showed the following main findings: (1) RhE is a sensitive model to detect irritant-induced cytotoxicity; (2) FTS model is a better model to detect irritant-induced IL-1α secretion; and (3) the secretion of IL-1α and IL-18 may distinguish chemical’s irritation and sensitization potential. Overall, the monolayer culture system accommodates the need of large high throughput primary screening, while RhE and FTS models address more functional endpoints such as barrier function and inflammatory response.

Our study used several novel techniques to produce RhE and FTS models. First, the organotypic skin constructs, produced by bio-printing technology, allowed for controlled spatial cell layering with consistent cellular composition, cellular distribution, and extracellular matrix (ECM) organization. Second, the use of fibrin gel as a bio-printing ink has shown advantages in long term (over 12 weeks) cultures by preventing contraction when compared to conventional collagen-based models (Boehnke et al., 2007; Sriram et al., 2015; Derr et al., 2019). This feature also allows for the topical application of compounds without leakage from the side of the well occurring. When measuring TEER values, a tissue surface coverage of less than 99.6% has previously been shown to cause an 80% drop in barrier function (Florin et al., 2005). The consistent and high TEER values, from both RhE and FTS models developed in this study, further emphasized that a proper bio-printing ink can provide a full surface area without shrinkage; this accommodates accurate TEER measurements which indicate barrier function integrity. Third, the bio-printed structure can accommodate different research needs by permitting custom printing of tissue onto various sizes of transwell inserts.

OECD test guidelines 431 and 439 use RhE as the *in vitro* model for skin corrosion and irritation tests (Co-operation and Development, 2013; OECD, 2015). In this study, we introduced a novel bio-technique to produce 96-well plate RhE and FTS models for HTS applications. The bio-fabricated RhE is capable of detecting corrosives, by applying raw material as suggested in OECD test guidelines 431 and 439 (Supplementary Figure 1). Due to the limited concentration (highest equaled 200 μM) of test chemicals in the compound library, some reported irritants (see global harmonized system (GHS) hazard information with H315 (indicating skin irritation) labeled in Supplementary Table 2) did not reduce viability in RhE. Also, most compounds that showed a reduction in tissue viability in RhE were not detected in FTS. Only methylrosaniline chloride showed disruption of tight junction through a decrease of TEER values in RhE model and a decrease of viability in both RhE and FTS models. This unexpected observation may be due to less of the compound being able to penetrate the dermis, which in turn causes fibroblast death. It also explains the greater concordance between viability and TEER endpoints in the RhE model over the FTS (Figure 5A). The endpoint of TEER values in the FTS appeared to be a more sensitive endpoint of skin corrosion or irritation rather than tissue viability. For example, TEER identified benzethonium as an irritant in the FTS, independent of cytotoxicity. However, TEER is very sensitive and may generate false positive irritation effects. Therefore, other readouts, such as tissue viability and IL-1α secretion level, should be considered in conjunction when studying TEER values.

Hexachlorophene is used as a topical anti-infective and anti-bacterial agent in soaps and toothpaste. It is also used in agriculture as a soil fungicide, plant bactericide, and acaricide (Fiege et al., 2000). Studies have shown that some patients tested with a concentration range of 0.3% to 6% transdermal patches (7–147 μM) were allergic to this substance (Schoppelrey et al., 1997). At a high concentration (200 μM), hexachlorophene dramatically increased the IL-1α secretion in the RhE and FTS models (Figure 4A). Further concentration-response testing in the FTS model, showed that IL-1α was not elevated at lower concentrations (12.5–50 μM), whereas IL-18 was elevated at these lower concentrations (Figure 7B). The IL-18 elevation indicated the sensitization potential of hexachlorophene which explained the allergic response in clinic.

Methylrosaniline chloride (also known as gentian violet) and methyl violet are the most frequently used topical skin agents among the triphenylmethane dyes (Torres et al., 2009). We have observed that both compounds elicited an increase in IL-18 secretion at the lowest concentration (12.5 μM), but not IL-1α when treated in the FTS model (Figures 7A,C). Interestingly, even though methylrosaniline differs from methyl violet by only a methyl group, it causes more severe tissue damage than the latter (Figures 6E,G). Methyl violet has been reported as a carcinogen and eye irritant in mice (Littlefield et al., 1985), as well as a few reports of contact sensitization to this dye (Bielicky and Novák, 1969; Bajaj and Gupta, 1986). The first report of methylrosaniline-caused allergy was as early as 1940 (Goldstein, 1940). At that time, 3% methylrosaniline solution was applied in an intertriginous space; meaning that the observed response could be due to toxicity or irritancy rather than a physical allergy. In 2009, there was a case report about irritant contact dermatitis caused by a methylrosaniline patch at the therapeutic concentration of 0.5% (Torres et al., 2009); this percentage is close to the 12.5 μM concentration which we observed a significant IL-18 elevation, but not IL-1α.

Based on the elevated IL-18 secretion from methyl violet and methylrosaniline treatment, both compounds could be considered sensitizers at low concentrations (less than 50 μM) and irritants at high concentrations. In addition, methylrosaniline’s sensitizing effect of increasing IL-18 were only observed in FTS, not in the RhE model (Figures 4C,D), suggesting that a more complex model will better characterize inflammatory response upon chemical treatment.

Fibroblasts play an important role in skin tissue morphogenesis, homeostasis, and various histopathological conditions (Sriram et al., 2015). Adding dermis to the tissue fits the need for *in vitro* skin tissue to mimic physiological architecture of human native skin. Dermal fibroblasts interact with keratinocytes through direct cell-cell communications, cell-matrix interactions, and secretion of growth factors and cytokines (Werner et al., 1992; Werner et al., 1994; Mueller and Fusenig, 2002). The heatmap in Figure 5B indicated that RhE was more responsive to IL-18 secretion, while FTS captured more dramatic changes in IL-1α secretion. Cell-cell signaling between epidermal and dermal cells influence toxicological effects of compounds, specifically cytokine secretions (Maas-Szabowski et al., 2001). When investigating with the selected compounds, our data confirmed that fibroblasts and keratinocytes have cell to cell communication; this modulates the keratinocytes’ response to environmental insults in a more physiologically relevant pattern. For example, methylrosaniline chloride and hexachlorophene generated a greater IL-1α secretion level in FTS than in RhE. We speculate that this is due to the dramatic increase of IL-1α secretion when in the presence of fibroblasts, and was therefore mediated by cell-cell signaling between the fibroblasts and keratinocytes.

During the production of FTS, we observed a drastic decrease in TEER values when using FTS after 11 days of tissue culture (data not shown). These lower TEER values suggested that the tight junction barrier formed between the keratinocytes and fibroblasts affected barrier function; this may be due to cell-cell interactions and signaling between both types of cells. Another possibility is the different medium used for FTS; using another type of medium introduced in a previous article (Sriram et al., 2018), the TEER values reached maximum at day 12 with a value of 1022.7 ± 246 Ω^∗^cm^2^ (*n* = 60) (data not shown). Therefore, the bio-printing technique for each assay must be optimized, since the FTS maturation can occur at different times when different media is being used.

Our study tested 46 prioritized compounds related to topical products on monolayer keratinocytes, RhE, and FTS models. Each platform holds the potential for identifying dermal hazards, but each of these assays need extensive confirmation testing to be able to rely on their predictive ability. The scientific community generally accepts using the endpoints of TEER, cell viability, and IL-1α for assessing irritation potential, and IL-18 secretion levels for assessing sensitization potential. The integrated measurement of barrier function, inflammatory response, and tissue damage, combined these readouts and provided a comprehensive way to evaluate irritation potential. This novel approach could be used as an evidence for hazard labeling in the Globally Harmonized System (GHS), pesticide registration in the United States Environmental Protection Agency (EPA), and consumer product regulation by U.S. Consumer Product Safety Commission (CPSC). We believe using human cells to generate bio-printed tissue is a quick and reliable method to model human skin in a high-throughput manner. This newly biofabricated skin tissue can be used for the safety profiling of topically applied compounds, as well as in the earlier stages of drug discovery. Eventually, the HTS biofabricated models described here will potentially be able to speed up the translation of new candidate therapeutics to the clinic and new consumer products to the market.

## Data Availability Statement

All datasets for this study are included in the article/Supplementary Material.

## Author Contributions

ZW, XL, MX, and MF designed the study. ZW, XL, MO, LZ, and MS executed the experiments. RH and NK select compounds and curate datasets. ZW, XL, MX, and MF wrote the manuscript. RH, AS, and NK edited the manuscript. All authors read and approved the final version of the manuscript.

## Disclaimer

The views expressed in this article are those of the authors and do not necessarily reflect the views or policies of the National Institute of Environmental Health Sciences, the National Center for Advancing Translational Sciences, National Institutes of Health, or the United States Government. Mention of trade names or commercial products does not constitute endorsement or recommendation for use.

## Conflict of Interest

The authors declare that the research was conducted in the absence of any commercial or financial relationships that could be construed as a potential conflict of interest.

## Supplementary Material

FIGURE S1 Assessment of RhE model for **(A)** corrosion and **(B)** irritation test of benchmark chemicals from OECD test guidelines. **(C)** Comparison of tissue viability treated by 1% DMSO (vehicle control) and 5% SDS. OECD test guideline 439 protocol suggested 1 h treatment with raw material followed by 14 time intensive washing steps. Viability was measured after a 2-day post-treatment incubation. Modified treatment plan in the current study is continuous 3-day treatment of compounds at a lower concentration without intensive washing steps.

TABLE S1 Cell viability of top 46 most toxic compounds in NKTC and NHEK cells measured by CellTiter-Glo and WST-8.

TABLE S2 Comparison of RhE and FTS constructs on viability and TEER upon treatment with the 46 most toxic compounds.

TABLE S3 Medium composition for bio-printed full-thickness skin construct and primary and secondary antibodies used in the study.

TABLE S4 Cell viability of all topical-use compounds in NKTC and NHEK cells measured by CellTiter-Glo and WST-8.

## Abbreviations

2D: two-dimensional
3D: three-dimensional
ALI: air-liquid interface
ATCC: American Tissue Culture Collection
CPSCPSC: Consumer Product Safety Commission
CTG-3D: CellTiter-Glo^®^ 3D
DMEM: Dulbecco’s Modified Eagle Medium
EU: European Union
FDA: Food and Drug Administration
FDM: fully supplemented dermal medium
FTS: full thickness skin
GA-1000: Gentamicin sulfate-Amphotericin
H&E: hematoxylin and eosin
HTS: high throughput screening
IHC: immunohistochemistry
IL-1 α: interleukin 1 alpha
IL-18: interleukin 18
KGM: keratinocyte growth media
KRT 10: keratin-10
MSD: Meso Scale Discovery
NCATS: National Center for Advancing Translational Sciences
NDF: neonatal human dermal fibroblast
NHEK: normal human epidermal keratinocytes NHEK/SVTERT3-5
NKTC: human primary neonatal keratinocytes
OECD: Organization for Economic Co-operation and Development
PBS: phosphate buffered saline
PBST: phosphate buffered saline with 0.05% Tween 20®
RhE: reconstructed human epidermis
SB: stratum basale
SC: stratum corneum
SD: standard deviation
SG: stratum granulosum
SP: stratum spinosum
TEER: transepithelial electrical resistance
Tox21: Toxicology in the 21st Century.
