# Supplementary figures and images for: Two-Dimensional Cellular and Three-Dimensional Bio-Printed Skin Models to Screen Topical-Use Compounds for Irritation Potential
Source: Front Bioeng Biotechnol. 2020 Feb 21;8:109. doi: 10.3389/fbioe.2020.00109 (PMC7046801; doi:10.3389/fbioe.2020.00109)

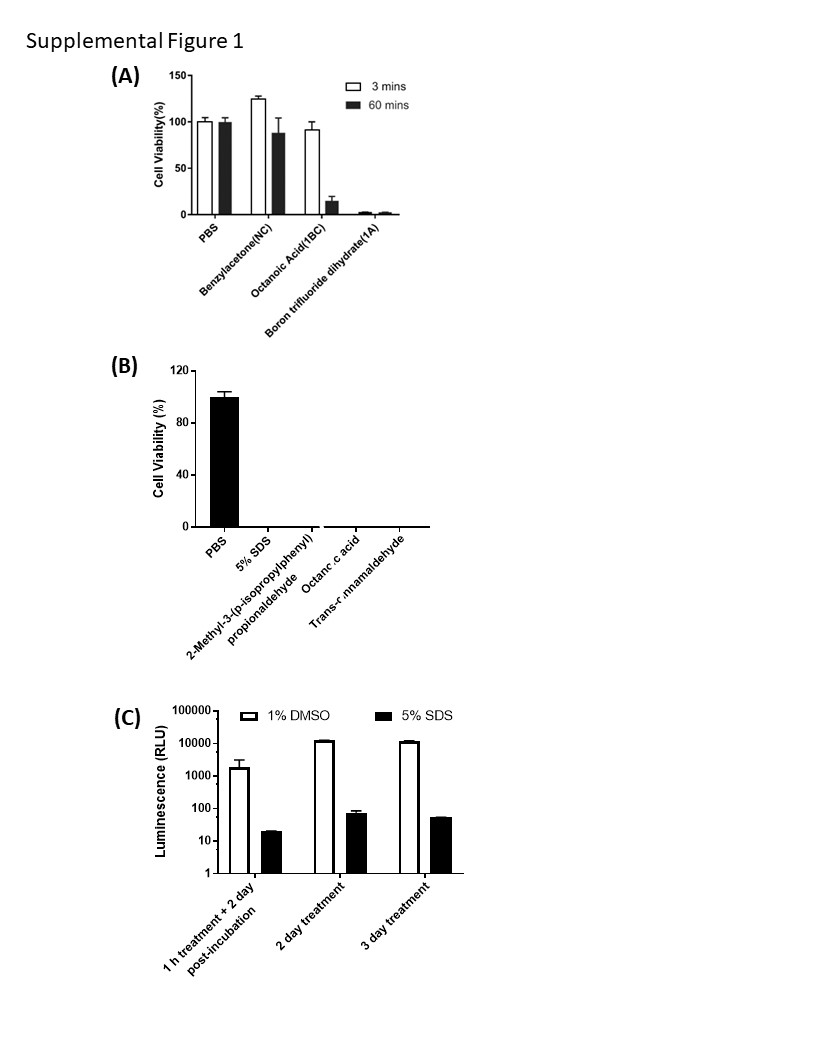

Supplement: FIGURE S1 — Assessment of RhE model for (A) corrosion and (B) irritation test of benchmark chemicals from OECD test guidelines. (C) Comparison of tissue viability treated by 1% DMSO (vehicle control) and 5% SDS. OECD test guideline 439 protocol suggested 1 h treatment with raw material followed by 14 time intensive washing steps. Viability was measured after a 2-day post-treatment incubation. Modified treatment plan in the current study is continuous 3-day treatment of compounds at a lower concentration without intensive washing steps. [file Image_1.JPEG]
