# Supplementary material for: Two-Dimensional Cellular and Three-Dimensional Bio-Printed Skin Models to Screen Topical-Use Compounds for Irritation Potential
Source: Front Bioeng Biotechnol. 2020 Feb 21;8:109. doi: 10.3389/fbioe.2020.00109 (PMC7046801; doi:10.3389/fbioe.2020.00109)
Supplement: TABLE S3 — Medium composition for bio-printed full-thickness skin construct and primary and secondary antibodies used in the study. [file Table_3.DOCX]

Supplemental Table 3. Medium composition for bio-printed vascularized full-thickness skin construct and primary and secondary antibodies used in the study.

| Media | Component | Source | Amount |
| --- | --- | --- | --- |
| iCell EndothelialCells Medium | VascuLife Basal Medium | Cellular Dynamics International(M1019) | 475 mL |
|  | rh VEGF |  | 0.5 mL |
|  | rh EGF |  | 0.5 mL |
|  | rh FGF basic |  | 0.5 mL |
|  | rh IGF-1 |  | 0.5 mL |
|  | Ascorbic Acid |  | 0.5 mL |
|  | Hydrocortisone Hemisuccinate |  | 0.5 mL |
|  | Heparin Sulfate |  | 0.5 mL |
|  | L-glutamine |  | 0.5 mL |
|  | iCell Endothelial Cells Medium Supplement |  | 50 mL |
|  | | | |
| Media | Component | Source |  |
| MEM Medium | MEM, alpha modification | ThermoFisher (Cat#32561102) | 500mL |
|  | N1 supplement | Sigma(Cat#N6530) | 5mL |
|  | Penicillin-streptomycin | ThermoFisher (Cat#15140122) | 5mL |
|  | GlutaMax | ThermoFisher (Cat#35050061) | 5mL |
|  | MEM Non-essential Amino Acid Solution | Sigma(Cat#M7145) | 5mL |
|  | THT |  | |
|  | -Taurine | Sigma(Cat#T0625) | 125 mg |
|  | -Hydrocortisone | Sigma(Cat#H0396) | 10 µg |
|  | -Triiodo-thyronin | Sigma(Cat#T5516) | 0.0065 µg |

| FDM-A | Component | Source | Stock Concentration | Amount |
| --- | --- | --- | --- | --- |
|  | iCell Medium |  |  | 100 mL |
|  | MEM Medium |  |  | 200 mL |
|  | Aprotinin | Sigma(Cat#A4529) | 0.075 UI/mL | 1.2 mL |
|  | Fetal Bovine Serum | ThermoFisher(Cat#A3840002) |  | 5 mL |
|  |  |  |  |  |

| FDM-B | Component | Source | Stock Concentration | Amount |
| --- | --- | --- | --- | --- |
|  | iCell Medium |  |  | 100 mL |
|  | MEM Medium |  |  | 200 mL |
|  | Aprotinin | Sigma(Cat#A4529) | 0.075 UI/mL | 1.2 mL |
|  | SITE+3 supplement | Sigma(Cat#S5295) |  | 3 mL |
|  | Calcium chloride | Sigma(Cat#21115) | 1mol/L | 0.33 mL |
|  | Fetal Bovine Serum | ThermoFisher(Cat#A3840002) |  | 5 mL |

| Antibody | Species reactivity | Specification | Dilution | Source |
| --- | --- | --- | --- | --- |
| Collagen-IV | Human, bovine | Rabbit polyclonal | 1:50 | Abcam(Cat # ab6586) |
| Desmoglein-1 | Human, mouse, rat | Rabbit monoclonal | 1:50 | Abcam(Cat # ab124798) |
| Filaggrin | Human | Mouse monoclonal | 1:50 | Thermo Fisher(Cat# MA5-13440 |
| Claudin1 | Human, rat, chicken | Rabbit polyclonal | 1:50 | Thermo Fisher(Cat# 51-9000) |
| Loricrin | Human, mouse | Rabbit polyclonal | 1:1000 | Biolegend(Cat# 905101) |
| KRT-10 | Human, dog, rat, mouse | Mouse monoclonal | 1:100 | Thermo Fisher(Cat#DE-K10) |
| Phalloidin |  | Alexa Fluor 647 | 1:40 | Thermo Fisher(Cat#A22287) |
| Secondary antibody |  | Alexa Fluor 488; Goat anti-mouse | 1:50 | Thermo Fisher(Cat#A-11001) |
| Secondary antibody |  | Alexa Fluor 594; Goat anti-rabbit | 1:50 | Thermo Fisher(Cat#A-11037) |
| Hoechest 33342 |  |  | 1:2000 | Thermo Fisher(Cat# 62249) |
